# Attenuation measurements on non-contrast-enhanced CT brain: a re-visit of their role in the diagnosis of cerebral venous thrombosis

**DOI:** 10.1007/s11604-025-01746-0

**Published:** 2025-02-18

**Authors:** Ahmed Abdrabou, Ahmed M. Osman, Noha M. Taha, Eman A. F. Darwish

**Affiliations:** Faculty of Medicine, Ain Shams University, Block 112, Building 103, Madinaty, Cairo, Egypt

**Keywords:** Cerebral venous thrombosis, Sinus attenuation, Hematocrit, Hounsfield unit, Computed tomography

## Abstract

**Purpose:**

To determine the diagnostic merit of using quantitative imaging parameters measured on non-contrast CT (NCCT) in the diagnosis of cerebral venous thrombosis (CVT).

**Materials and methods:**

43 patients with CVT and 30 age and sex-matched controls were enrolled in this retrospective study. Two blinded neuroradiologists independently assessed the NCCT scans for direct and indirect signs of CVT. Absolute attenuation values of thrombosed and non-thrombosed sinuses were measured and the ratio of sinus attenuation to patient’s hematocrit (H/H ratio) was calculated for each case. The attenuation value of the internal carotid artery was also measured to calculate the veno-arterial (V/A) difference. Inter-reader agreement was evaluated and all measurements were compared between patient and control groups.

**Results:**

The sensitivity and specificity of the dense sinus sign for diagnosis of CVT ranged from 72 to 79% and from 83 to 87% respectively. Sinus attenuation, H/H ratio and V/A difference were significantly higher in the CVT group than in the control group (*P* < 0.0001). An attenuation threshold value of > 55 HU yielded a sensitivity of 86% and a specificity of 90% and had the largest AUC of 0.89 (95%CI 0.795–0.951) among all stand-alone parameters, while the combination of an attenuation value greater than 55, or a V/A difference greater than 22, yielded the best diagnostic performance among all the parameters with sensitivity and specificity at values of 86% and a 100% respectively and an AUC of 0.93 (95%CI 0.846 to 0.977).

**Conclusions:**

Overall, the analysis of quantitative parameters resulted in a notable upgrade in the accuracy of the diagnosis of CVT over visual inspection alone. Their high specificities make them reliable markers of CVT, yet their relatively lower sensitivities may indicate a need to perform further studies to safely eliminate the possibility of CVT in highly suspicious cases.

Trial registration number: FMASU R 10/2020/2021.

## Introduction

Cerebral venous thrombosis (CVT) is a potentially life-threatening condition that is currently thought to be more prevalent than the previously reported annual incidence of 2 to 7 cases per 1,000,000. Prompt detection of CVT is critical as delayed treatment may result in irreversible brain damage, permanent disability or even death [1]. Given its widespread availability, non-contrast computed tomography (NCCT) remains the first-line imaging modality for the often ambiguous neurological symptoms that accompany CVT. Though of limited sensitivity and specificity, an increase in the attenuation of the obstructed sinus is the only direct sign of CVT on NCCT [2]. Since the degree of attenuation of blood is dependent on the protein component of hemoglobin, conditions characterized by hemoconcentration and increased hematocrit (HCT) levels such as polycythemia, dehydration, and young age may lead to a false diagnosis of CVT [2]. Alternatively, increased sinus attenuation is often subtle and easily missed on qualitative evaluation of the unenhanced scans [3]. In recent years a number of studies have suggested that the sensitivity and specificity of unenhanced CT for the diagnosis of CVT can be improved by measuring the attenuation of the suspicious sinus and comparing it to the hematocrit level of the patient [1–5]. The aim of this study was to evaluate the value of quantitative parameters including absolute sinus attenuation, the ratio of sinus attenuation to serum hematocrit and the difference between sinovenous attenuation and arterial attenuation in differentiating patients with and without CVT.

## Materials and methods

### Patients

We searched our electronic database for patients with CVT who were admitted to our hospital in the period from March 2022 to March 2023. Patients were considered eligible if they fulfilled the following criteria: (1)18 years or older, (2) had an NCCT performed on admission followed by either a CT venogram (CTV) or a magnetic resonance venogram (MRV) that established the diagnosis of CVT. Additionally, adults who had an NCCT performed for neurological symptoms, yet whose subsequent CTV or MRV confirmed the absence of CVT, were chosen as controls from our database. Subjects were excluded from the study if their non-contrast CT displayed any artifact (eg motion artifact, beam hardening artifact, or metal artifact) or pathology (eg skull fracture, operative lesion or intracranial hemorrhage even if it was due to underlying venous thrombosis) that obscured the venous sinuses and thus interfered with or prevented accurate measurement of sinus attenuation. Cases that received intravenous iodinated contrast for any reason within 3 days prior to the non-enhanced CT brain were also excluded as were patients with isolated cortical or deep venous thrombosis. All participants had to have a complete blood count performed within 24 h of the CT that clearly stated the hematocrit level. A total of 43 patients and 30 controls met our criteria and were included in the study.

### Image acquisition

The CT examinations were performed on 64-slice Optima™ CT660 scanner GE Healthcare, Milwaukee, WI. Image acquisition parameters for all CT examinations are detailed in Table 1

**Table 1:** Scanning parameters for CT examinations

	Scan type	Beam collimation	Detector rows	Detector configuration	Scan FOV	Pitch	Speed (mm/rot)	Rotation time (s)	KV	AEC type	mA range	Manual mA	Slice thickness (mm)	Interval (mm)
Brain	Helical	20	32	32 × 0.625	head	0.531	10.60	0.5	120	Smart mA	130–440	210	5	3
Venography	Helical	40	64	64 × 0.625	Large body	0.516	20.60	0.6	100	Smart mA	250–460	422	0.625	0.312

AEC: Automatic exposure control; FOV: Field of view

For all CT examinations, scanning started at the bottom of C1 and commenced at the top of the vertex. Coronal and sagittal images were reformatted with a thickness of 3mm and an interval of 1.5mm CTV examinations were performed using the GE Bolus Tracking Feature—“Smart Prep” after injection of 90 ml of Iohexol 300 mg/mL at an injection rate of 4ml/s + 40 ml NaCl flush. Axial, sagittal, and coronal thick-slab maximum intensity projection images (MIPs) through the entire head were generated with a slice thickness of 10mm and an interval of 2.5 mm.

### Image analysis

Anonymous non-enhanced CTs were presented separately to two neuroradiologists with 5 and 7 years of experience. Both were blinded to the patient versus control status of the study and to clinical data. Each CT was evaluated for indirect (edema and/or hemorrhage) and direct (hyper-attenuating sinus) signs of CVT. The superior sagittal sinus, left and right transverse sinuses, left and right sigmoid sinuses and the straight sinus were assessed systematically. If readers suspected CVT, they were asked to measure the attenuation of the affected sinus by placing three non-overlapping regions of interest (ROIs) within the area of the sinus they felt was the densest on axial or reformatted images. Each ROI was manually drawn making it as large as possible without involving the sinus wall, adjacent skull or surrounding parenchyma. The mean of the three measurements from the affected sinus was calculated and the average absolute attenuation value was recorded in Hounsfield units (HU). In studies where no CVT was detected readers obtained measurements from three ROIs, each placed within a different, randomly selected sinus, provided it could be reliably discerned from the adjacent brain parenchyma, and the mean attenuation value was noted. To measure the venoarterial difference, two ROIs of interest were placed, one in each internal carotid artery (ICA) at a non-calcified segment of its intracranial portion. The mean ICA attenuation was determined and subtracted from the attenuation value of the thrombosed sinus in cases with CVT or from the attenuation value of the normal sinuses in cases without CVT. For all cases, the ratio of sinus attenuation to the hematocrit (H:H ratio) was calculated by dividing the absolute attenuation value of the thrombosed or normal sinus with the hematocrit.

### Statistical analysis

Statistical analysis was performed using MedCalc statistical software for Windows, version 22.009 (MedCalc Software, Ostend, Belgium). Data for continuous variables were expressed as median, interquartile range and range and as both number and percentage for categorical data. The agreement between readers was evaluated using the intraclass correlation coefficient (ICC) and Cohen’s Kappa coefficient for quantitative and qualitative variables respectively. All comparisons between patient and control groups were carried out by Mann–Whitney U-test. Receiver operator characteristic (ROC) curve analysis was performed to determine optimum threshold values for all variables and their diagnostic performance in distinguishing the different groups. The diagnostic performance of all variables was evaluated in terms of sensitivity, specificity, positive predictive value (PPV), negative predictive value (NPV), and area under the ROC curve (AUC). To reduce the effect of outliers, the averaged values of the two readers were used in this part of the analysis. For all tests, all P values were two-tailed and a P-value < 0.05 was considered significant.

## Results

**Fig. 1:**
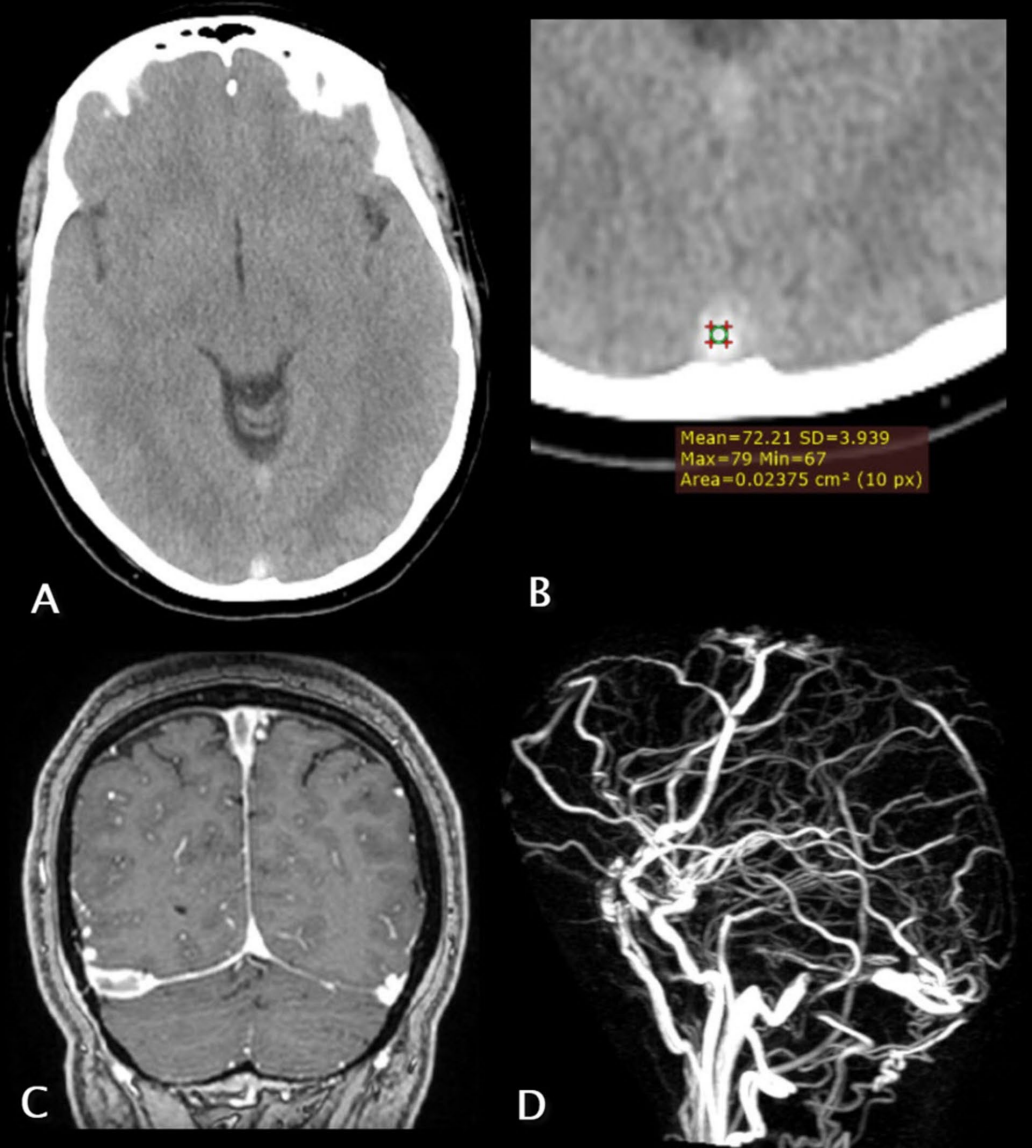
**A**–**D** Panels(**A**) and (**B**) are axial cuts of a non-contrast CT study of a 37 years old female patient who came to the emergency room complaining of headache and nausea while panels **C** and **D** are magnetic resonance images of the patient which were performed on the same day following the CT. Panel (**A**) revealed a hyperdense inferior segment of the superior sagittal sinus (SSS) and panel (**B**) shows that the measured density inside the sinus was about 72.4 HU which was significantly high. Panel (**C**) is a coronal contrast-enhanced MR image which revealed a filling defect at the superior sagittal and right transverse sinuses (empty delta sign) confirming the diagnosis of sinus thrombosis. Panel (**D**) is 3-dimensional reconstructed MRV image which showed the absence of superior sagittal and right transverse sinus and the presence of numerous tiny crowded vessels in the background presumed to be dilated collateral channels

**Fig. 2:**
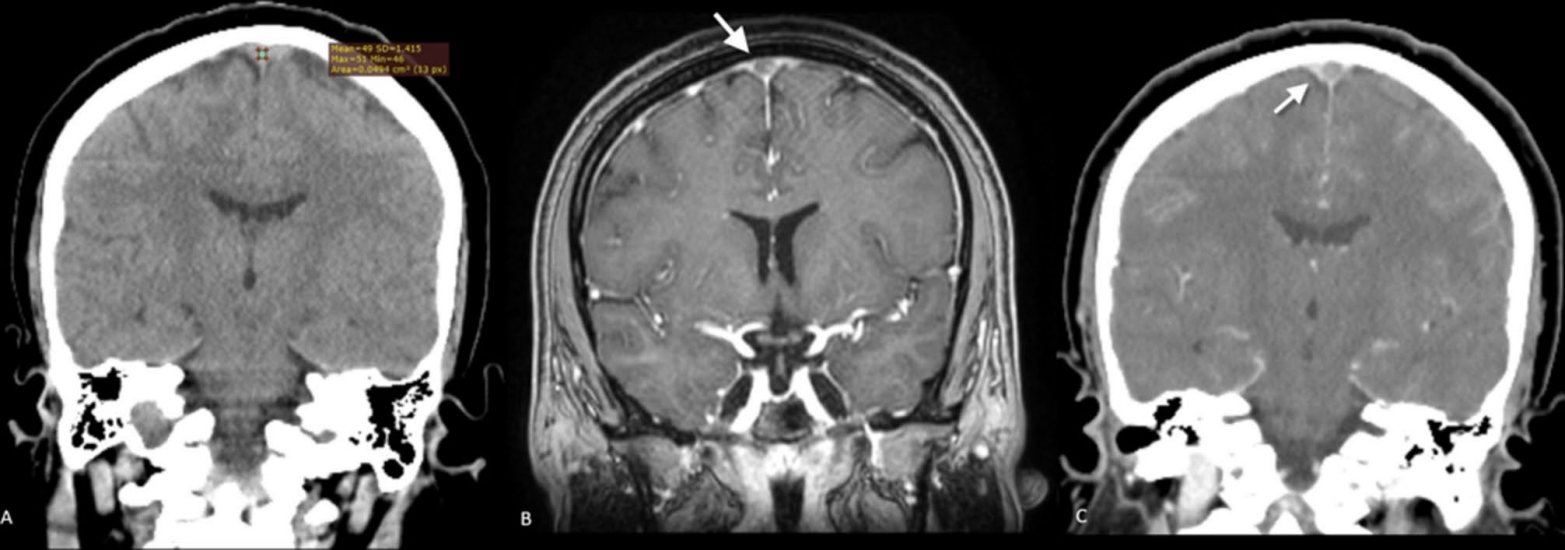
**A**–**C** Superior sagittal sinus (SSS) of a 36 year-old male appeared normal on non-contrast computed tomography with an average attenuation of 49 HU (**A**) and was interpreted as normal. However, contrast-enhanced CT in coronal (**B**) and CT venography in sagittal (**C**) reconstruction showed a well-defined filling defect within the SSS (arrows) consistent with thrombosis of the SSS. As the patient’s hematocrit was within normal range and the patient presented only by headache, a possible explanation for the false negative measurements may be a significant time interval between the onset of symptoms and imaging, by which time the thrombus elicited lower attenuation values

**Fig. 3:**
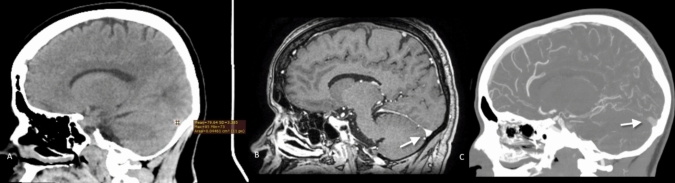
A–C The torcula of 30 year-old female appeared hyperattenuating on non-contrast computed tomography with an average attenuation of 79 HU (**A**) and was interpreted as thrombosed. However, contrast-enhanced CT in sagittal (**B**) and CT venography in sagittal (**C**) reconstruction showed patency of the torcula. This false positive result is likely attributed to the patient’s elevated hematocrit level of 50%

A total of 43 patients with an established diagnosis of CVT and 30 controls who were confirmed not to have CVT were enrolled in this study. Details of the demographic data and clinical characteristics of both groups are outlined in Table 2 and examples from our cases can be seen in Figs. 1, 2, 3.

**Table 2:** demographic and clinical data for patients and controls

Parameter	Patients	Controls
Sex, number (%)		
•Male	13 (30.2)	22 (73.3)
•Female	30 (69.8)	8 (26.7)
Age (years)		
•Median	39	49.5
•IQR	30–49	34–61
•Range	18–93	20–86
Risk factors, number (%)		
•No risk factors	15 (34.9)	30 (100)
•Oral contraceptive pills	8 (18.6)	–
•Post-partum	6 (14)	–
•Thrombophilia	4 (9.3)	–
•Inflammatory/autoimmune disease	4 (9.3)	–
•Cancer	3 (7)	–
•Intracranial infection	2 (4.7)	–
•End stage renal disease	1 (2.3)	–
Complaint, number (%)		
•Headache ± other symptoms	26 (60.5)	14 (46.7)
•Convulsions	7 (16.3)	1(3.3)
•Disturbed conscious level	6 (13.9)	8 (26.7)
•Trauma	–	4 (13.3)
•Dizziness	–	3(10)
•Hemiparesis	4 (9.3)	–
Hematocrit %		
•Mean ± SD	40.8 ± 6.31%	43.6 ± 4.8%
•Median	42%	45%
•Range	30–52%	34–50%
•IQR	35–46%	41–48%
Thrombosed sinus, number (%)		
•Unilateral transverse sinus	16 (37.2)	
•SSS	14 (32.6)	
•SSS and unilateral transverse sinus	5 (11.6)	
•Unilateral transverse and sigmoid sinuses	3 (7)	
•Vein of Galen and straight sinus	2 (4.7)	
•Straight sinus and unilateral transverse sinus	2 (4.7)	
•SSS and bilateral transverse sinuses	1 (2.3)	
Parenchymal findings, number (%)		
•Edema and hemorrhage	5 (11.6)	–
•Edema only	3 (7)	–

Regarding the diagnosis of CVT based on the qualitative evaluation of sinus density, a strong agreement was found between the two readers with a kappa value of 0.67 (95% CI 0.50 to 0.84). The diagnostic performance of the hyperdense sinus sign in the diagnosis of CVT can be seen in Table 3.

**Table 3:** Diagnostic performance of hyperdense sinus sign in the diagnosis of CVT

	Sensitivity (95% CI)	Specificity (95% CI)	Positive predictive value (95% CI)	Negative predictive value (95% CI)	Positive likelihood ratio (95% CI)	Negative likelihood ratio (95% CI)	Area under curve (95% CI)	Accuracy (95% CI)
Reader 1	79.1% (64.0%–90.0%)	86.7% (69.3%–96.2%)	89.5% (77.1%–95.5%)	74.3% (61.4%–84.0%)	5.93 (2.4–15.0)	0.24 (0.13–0.44)	0.83 (0.72–0.91)	82.2% (71.5%–90.2%)
Reader 2	72.1% (56.3%–84.7%)	83.3% (65.3%–94.4%)	86.1% (73.2%–93.4%)	67.6% (55.7%–77.6%)	4.33 (1.9–9.84)	0.34 (0.2–0.56)	0.78 (0.67–0.87)	76.7% (65.4%–85.8%)

CI: confidence interval

The agreement between the two readers with respect to the quantitative variables was very high, with an intraclass correlation coefficient of 0.94 (95% CI 0.91 to 0.97) for attenuation, 0.96 (95% CI 0.94 to 0.98) for H:H ratio, and 0.93 (95% CI 0.88–0.95) for the veno-arterial difference. For the 2 readers, the sinus attenuation, H/H ratio and veno-arterial difference were all significantly higher in the CVT group as compared to the control group (P < 0.0001) (Table 4).

**Table 4:** The quantitative parameters for patient and control groups

Attenuation (HU)		Median	IQR	Range	*P* value
Reader 1	Control	51	46–53	32–64	
	CVT	70	65.3–75.8	32–84	< 0.0001
Reader 2	Control CVT	50 72	46–54 58.3–75	36–67 30–81	< 0.0001
Average	Control CVT	50.5 71	47.5–53 57.6–76.3	34–65.5 31–82	< 0.0001
Attenuation/hematocrit ratio (H/H)					
Reader 1	Control	1.1	1–1.3	0.7–1.6	
	CVT	1.7	1.5–2	0.8–2.5	< 0.0001
Reader 2	Control CVT	1.1 1.7	1–1.3 1.4–1.9	0.8–1.7 0.8–2.5	< 0.0001
Veno-arterial difference					
Reader 1	Control	11.5	6–16	0–25	
	CVT	30	23.3–34.8	− 7–43	< 0.0001
Reader 2	Control	11	6–16	0–26	
	CVT	31	16.3–35	− 9–45	< 0.0001

CVT: cerebral venous thrombosis; IQR: interquartile range

No significant correlation was found between attenuation and hematocrit in both patients (*r*_*s* =_ 0.21, P = 0.18) and controls (*r*_*s*_ = 0.21, P = 0.27).

**Fig. 4:**
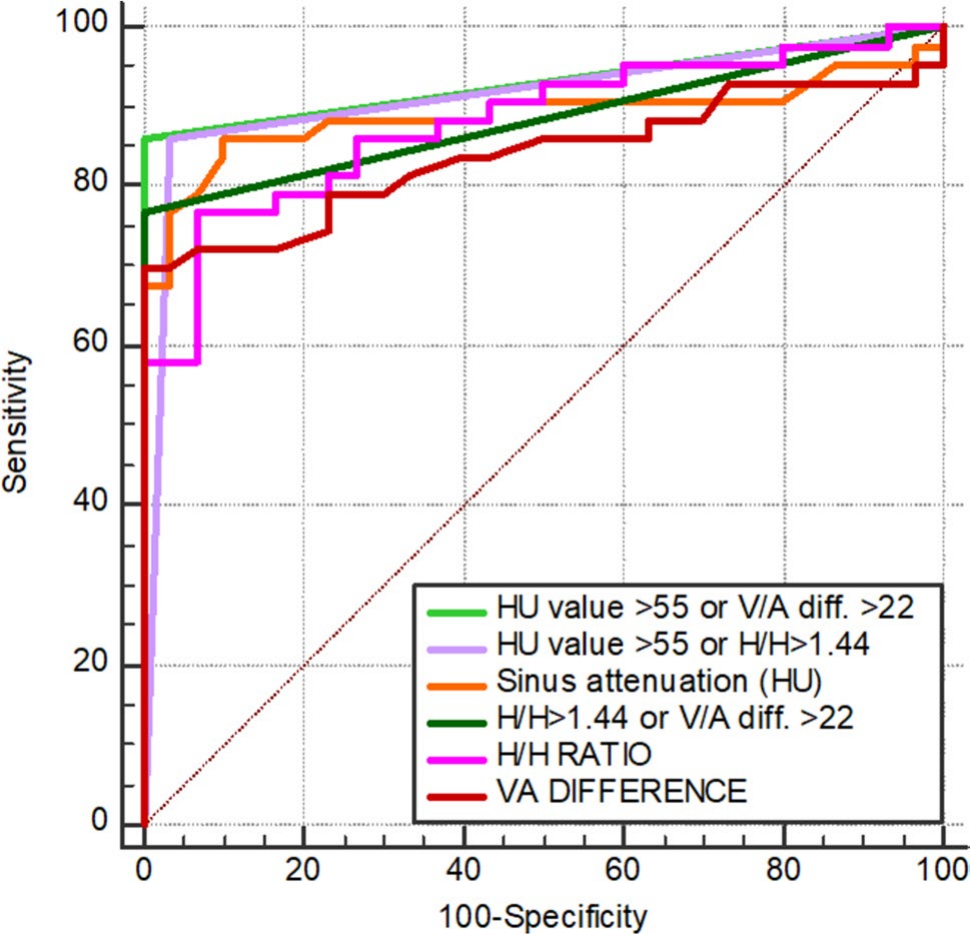
Comparison of the ROC curves for all parameters with regard to the diagnosis of CVT

ROC analyses were performed to determine optimal threshold values for all variables (Table 5). An optimal attenuation cut-off value of > 55 HU had 86.1% sensitivity and 90% specificity for CVT, with the largest AUC of 0.890 (95%CI 0.795–0.951) among all stand-alone parameters. The adjunctive utilization of a sinus attenuation value of greater than 55, or a V/A difference greater than 22, yielded the best diagnostic performance among all the parameters with sensitivity and specificity at values of 86% and 100%, respectively, and an AUC of 0.930 ( 95%CI 0.846 to 0.977). Pair-wise comparison of the ROC curves for all parameters revealed that the AUC of sinus attenuation > 55 or V/A diff. > 22 was significantly larger than that of H/H > 1.44 or V/A diff. > 22 (P = 0.038) and that of the veno-arterial difference (P = 0.002). The AUCs of the combined use of sinus attenuation > 55 or H/H > 1.44 and sinus attenuation alone were also both significantly greater than the AUC of the veno-arterial difference at P values of 0.018, and 0.016 respectively, otherwise no significant difference was seen between any of the other parameters (Table 6 and Fig. 4).

**Table 5:** Comparison of optimal threshold values for all variables as regards the accurate diagnosis of CVT

	Cut-off	Sensitivity (%)	Specificity (%)	AUC	95% CI	PPV (%)	NPV (%)	*P* value
Attenuation (HU)	> 55	86.1	90	0.890	0.795 to 0.951	92.5	81.8	< 0.0001
H/H ratio	> 1.44	76.7	93.3	0.881	0.784 to 0.945	94.3	73.7	< 0.0001
V/A difference	> 22	69.8	100	0.840	0.735 to 0.915	100	69.8	< 0.0001
HU > 55 or H/H > 1.44		86	96.7	0.914	0.824 to 0.967	97.4	82.9	< 0.0001
HU > 55 or V/A diff. > 22		86	100	0.930	0.846 to 0.977	100	83.3	< 0.0001
H/H > 1.44 or V/A diff. > 22		76.7	100	0.884	0.787 to 0.947	100	75	< 0.0001

AUC: area under curve; CI: confidence interval; H/H: ratio of sinus attenuation to hematocrit; HU: attenuation measured in Hounsfield units; PPV: positive predictive value; NPV: negative predictive value; V/A diff: veno-arterial difference

**Table 6:** Pair-wise comparisons of the area under ROC curves for all variables

	Attenuation		H/H ratio		HU > 55 or H/H > 1.44		V/A difference		HU > 55 or V/A diff. > 22		H/H > 1.44 or V/A diff. > 22	
	Diff	*P*- value	Diff	*P*- value	Diff	*P*-value	Diff	*P*-value	Diff	*P*-value	Diff	*P*-value
Attenuation (HU)	–	–	0.009	0.753	0.024	0.374	0.050	0.016	0.040	0.076	0.006	0.843
H/H ratio	–	–	–	–	0.032	0.247	0.042	0.176	0.049	0.092	0.002	0.932
HU > 55 or H/H > 1.44	–	–	–	–	–	–	0.074	0.018	0.017	0.317	0.029	0.285
V/A difference	–	–	–	–	–	–	–	–	0.091	0.002	0.044	0.147
HU > 55 or V/A diff. > 22		–	–	–	–	–	–	–	–	–	0.047	0.038

Diff.: difference between areas; H/H: ratio of sinus attenuation to hematocrit; HU: attenuation measured in Hounsfield units; V/A diff: veno-arterial difference

## Discussion

The diagnosis of CVT on a non-contrast head CT depends mainly upon the subjective recognition of the “hyperdense sinus sign,” which is observed when a thrombus has recently formed within the sinus lumen. This observable increase in attenuation results from clot retraction which eliminates water and subsequently increases the concentration of red blood cells and hemoglobin leading to an increase in the density of the thrombosed sinus. While the sensitivities of 79.1% and 72.1% reported by readers 1 and 2 respectively for the hyperdense sinus sign in the diagnosis of CVT in this study are higher than those reported by previous studies, a fact likely attributed to the improvements made to multidetector CT technology, they still remain inadequate for safely excluding the diagnosis of sinus thrombosis especially in the absence of the secondary signs of CVT [3, 5–8]. Interestingly the specificities reported by both readers were lower than those previously described [3, 5]. Although both readers were blinded to all clinical and imaging data, they knew the purpose of this study which could in part at least explain these results. Furthermore, the location of the thrombus, whether in the superficial venous sinuses or the deep cerebral veins may explain the variable results as Linn et al., emphasized that the diagnostic performance of qualitative density evaluation was superior in deep venous system thrombosis as compared to sinus thrombosis [7].

While an increase in sinus attenuation may often be too subtle to be recognized visually, a discernible elevation of sinus attenuation may also be fallacious. Therefore, realistic measurements of sinus attenuation may offer a more reliable method for the diagnosis of CVT [9]. In this study, median attenuation values of 70 and 72 HU generated by readers 1 and 2 respectively for thrombosed sinuses were significantly higher than the median attenuation values of 50 and 51 HU found by readers 1 and 2 respectively in non-thrombosed sinuses. Although the individual values differ, a consistent pattern was described by several previous studies [1–3, 5, 9]. In spite of these apparently promising results, the presence of overlap in the attenuation values of thrombosed and normal sinuses may impair the reliability of this method in everyday clinical practice. Based on our results a sinus attenuation threshold value of 55 HU yielded a sensitivity and specificity of 86.1% and 90%, respectively, for distinguishing patients with CVT from those without CVT. Our results differed from those of older studies by Buyck et al*.*, and Avesnik et al., who suggested higher cut-off values of 62 HU and 64 HU respectively with a better diagnostic performance [2, 9]. Their small sample sizes, however, may limit the trustworthiness of their findings. In fact in a more recent study also by Buyck et al*.*, on almost 300 patients, they found that their prespecified attenuation threshold of 62 HU did not attain the previously reported sensitivity and specificity levels [5].

Relying on absolute attenuation values may yield inaccurate results in patients with abnormal hematocrit (e.g. in patients with polycythemia, anemic patients or very young children) which is known to directly impact blood attenuation levels [10]. Normalization of sinus attenuation measurement as regards the HCT level may prove beneficial in such scenarios as it corrects for the abnormal HCT thus diminishing the risk for a false positive or negative analysis of sinus attenuation values [2]. Nevertheless, optimal cut-off values for the H/H ratio differed widely across different studies as did their diagnostic performances. Comparable to the H/H cut-off value of 1.8 suggested by Black et al., Besachio et al. reported an optimal H/H cut-off value of 1.7 with a sensitivity and specificity of 63% and 96% respectively [1, 4]. We found that a lower cut-off value of 1.44 yielded a sensitivity and specificity of about 77% and 93% respectively. This was closer to the cut-value of 1.5 reported by Buyck et al. yet they reported a far superior diagnostic performance with a sensitivity of 95%, specificity of 100%, and accuracy of 97.5% [2]. Interestingly however, this diagnostic performance could not be maintained when an H/H cut-off value of 1.5 was applied to a larger cohort of patients by the same author in a later study providing a sensitivity and specificity of only 74% and 70% respectively [5]. Differences in HCT may explain the resultant differences in the H/H values across different studies [1]. Since HCT is known to vary according to race, it is not surprising that our study, performed on a cohort of Egyptian individuals yielded different values [11]. Similar to Besachio et al., we demonstrated that the joint use of sinus attenuation and H/H ratio yielded a better diagnostic performance than that provided by either parameter alone, nevertheless the difference among them did not reach statistical significance indicating that in an emergency condition with no available lab results, measurement of absolute sinus attenuation can be used with a comparable degree of diagnostic confidence [1].

Consistent with the results of Besachio et al., we found that the veno-arterial difference was significantly greater in the thrombosed group than in the control group yet no agreement was found regarding its diagnostic performance [1]. In comparison to a veno-arterial difference threshold of greater than 15 HU, determined by Besachio et al., which provided a sensitivity and specificity of 80 and 94% respectively in the identification of CVT, we found that a threshold value of greater than 22 HU provided a much lower sensitivity of 69% but an optimum specificity of a 100% [1]. In fact, in contradistinction to Besachio et al., we found that veno-arterial difference was the most useful parameter when paired with sinus attenuation measurement which resulted in no loss to sensitivity provided by attenuation measurement alone but increased specificity, PPV and NPV up to 100%, 100% and 83.3% respectively [1]. This is valuable in an acute setting as the veno-arterial measurement can be easily obtained from any technically satisfactory study as opposed to the H/H ratio which requires additional lab testing. It is important to note that despite the apparent improvement in performance parameters when combining sinus attenuation with V/A difference over direct attenuation measurement alone, statistical tests reveal no significant difference in their AUCs suggesting that using absolute attenuation alone could be just as reliable as using it in conjunction with V/A difference. Nevertheless, this needs to be verified by larger prospective studies.

Finally, a point worth highlighting is that be it a venous attenuation value greater than 55, an H/H greater than 1.44, or a difference greater than 22 HU between venous and arterial attenuation, their high specificities indicates that with such readings the likelihood of true CVT is very high, possibly eliminating the need for further imaging in an appropriate clinical setting. Their relatively lower sensitivities, however, mean that further imaging studies such as CTV, or MRV may need to be performed to exclude sinus thrombosis in cases with a high index of clinical suspicion.

Limitations of this study include its retrospective design, which though we believe was most suitable due to the infrequency of CVT, may have resulted in sampling bias. Readers were not blinded to the parenchymal signs of CVT which when present could have led to bias during qualitative and quantitative assessment of sinus attenuation. Partial volume effects resulting from the close proximity of the skull bones to the venous sinus could have resulted in spurious hyper-attenuation of the sinus. We did not take into account the time elapsed from the onset of symptoms to the acquisition of the CT which may have influenced our results as the density of the thrombus is known to change over time. General guidelines were applied but precise standardization of measurements was not attained, and the exact location of ROI was left to the judgment of the readers which could affect the reliability of our results. Also the relatively small study cohort may restrict the dependability of the optimum cut-off values of the various quantitative parameters for the accurate diagnosis of CVT which need to be validated by large prospective multicenter trials.

In conclusion***,*** NCCT is the primary imaging technique for patients presenting with acute non-specific neurological symptoms, particularly so in impoverished regions where resources are limited. Therefore, any finding that may improve the capability of this modality to make a more accurate diagnosis is welcome. We found that absolute sinus attenuation, normalized ratio of sinus attenuation to hematocrit and veno-arterial difference are easily obtained quantitative variables that substantially enhanced the ability of radiologists to make an accurate diagnosis of CVT on NCCT as compared to visual assessment alone. Additionally, the use of quantitative parameters can help determine the need for additional confirmatory CT or MR imaging studies, thereby decreasing unnecessary imaging and enhancing efficient utilization of resources.

## Authorcontributions

All authors contributed to the study’s conception and design. Material preparation, data collection and analysis were performed by [Ahmed Abdrabou], [Ahmed M. Othman], [Noha M. Taha]. The first draft of the manuscript was written by [Eman A.F. Darwish] and all authors commented on previous versions of the manuscript. All authors read and approved the final manuscript. The authors confirm sole submission to Japanese Journal of Radiology. They all confirm that the article is not under consideration for publication elsewhere. All authors declare no subject overlap with previously published work.

## Abbreviations

NCCT: Non-contrast computed tomography
CVT: Cerebral venous thrombosis
H/H ratio: Ratio of sinus attenuation to hematocrit
HU: Hounsfield unit
AUC: Area under curve
V/A diff.: Veno-arterial difference
CI: Confidence interval
HCT: Hematocrit
CTV: Computed tomography venogram
MRV: Magnetic resonance venography
ROIs: Regions of interest
ICA: Internal carotid artery
ICC: Intraclass correlation coefficient
ROC: Receiver operator characteristic
PPV: Positive predictive value
NPV: Negative predictive value
SSS: Superior sagittal sinus
IQR: Interquartile range
